# Does stigma influence intentions to seek mental health care? A study among adults attending University in Ghana

**DOI:** 10.1371/journal.pmen.0000378

**Published:** 2025-08-20

**Authors:** Abigail Esinam Adade, DeGraft Nana Agyei, Evans K.S. Nyarko, Adote Anum, Rachel Yamson, Vivian Afi Dzokoto

**Affiliations:** 1 Department of Psychology, Virginia Commonwealth University, Richmond, Virginia, United States of America; 2 Department of Psychology, University of Ghana, Accra, Ghana; 3 Department of Psychology, The College of Wooster, Wooster, Ohio, United States of America; University of Sousse Faculty of Medicine of Sousse: Universite de Sousse Faculte de Medecine de Sousse, TUNISIA

## Abstract

We explored the factors influencing attitudes of help-seeking behavior and counseling intentions. Four hundred and forty (440) Ghanaian students from two public universities were conveniently sampled for this study. Data were collected through a cross-sectional survey using standardized questionnaires, including the Intention to Seek Counseling Inventory, Inventory of Attitudes Toward Seeking Mental Health Services, Attitudes Toward Seeking Professional Psychological Help Scale, Self-Stigma of Seeking Help Scale, and the Stigma Scale for Receiving Social Support. The data obtained were analyzed using multiple regressions. Our results revealed that self-stigma was significantly associated with attitudes toward seeking help from a psychologist, while social stigma was significantly associated with attitudes toward seeking help from a mental health service provider. Self-stigma, but not social stigma, moderated the relationship between attitudes toward seeking professional psychological help and intentions to seek counseling. This suggests that when self-stigma is high, attitudes to seek professional psychological help become less impactful in driving intentions to seek counseling. Interventions should focus on reducing self-stigma and empowering adults to overcome their internalized negative attitudes toward mental disorders, ultimately encouraging them to seek mental health care.

## Introduction

The prevalence of mental disorders has become a significant global public health crisis [1]. Approximately 970 million people worldwide are living with various mental health conditions, with the majority residing in low- and middle-income countries (LMICs) [2]. In Africa, over 116 million people live with mental health conditions, creating substantial economic and social burdens on the continent [2,3]. The high prevalence of mental disorders also hinders sustainable growth and development, negatively impacting overall well-being, particularly among adults who make up a substantial part of the labor force [3,4].

Despite the concerning rates and challenges associated with mental health conditions, many individuals hold negative attitudes and intentions toward seeking help [5]. Attitudes toward seeking help can be viewed through various lenses, including how individuals assess their emotional responses, openness, and perspectives on pursuing or avoiding professional psychological support [6]. On the other hand, intentions to seek help demonstrate an individual’s plan or willingness to actively address mental health challenges and utilize mental health services [7]. The relationship between attitudes and help-seeking intentions is crucial, as attitudes can influence the motivational factors driving these intentions [7–9]. Notably, individuals with positive attitudes toward mental health are more likely to use psychological services [10].

Several factors influence an individual’s attitude and intentions toward seeking psychological help. These factors include limited access to support services [11], adherence to masculinity norms [12], mental health illiteracy [13], and concerns about confidentiality [14]. Stigma is another significant factor contributing to reluctance in seeking psychological help [15]. It refers to exclusion, rejection, blame, or devaluation due to negative judgments about an individual or group [16]. In Ghana, Tawiah et al. identified three types of mental health stigma: social, economic, and psychological [17]. According to their study, social stigma is associated with blame and mockery from family members; economic stigma relates to financial limitations and unemployment; and psychological stigma encompasses internalized shame and feelings of inadequacy. Corrigan and Rao also described three forms of stigma: institutional, social, and self [18]. Institutional stigma arises from policies that limit opportunities for stigmatized groups, social stigma involves negative societal beliefs about mental disorders, and self-stigma entails the internalization and acceptance of societal stereotypes, leading individuals to apply these negative perceptions to their mental health issues [18].

In collectivist cultures, social stigma is heightened as individuals with mental illness are labeled as weak, which impedes help-seeking [19,20]. Supporting this, research has shown that White British individuals exhibit lower stigma and greater acceptance of mental health issues compared to South Asians, who tend to have stronger supernatural beliefs [21]. Similarly, public stigma has been reported to hinder help-seeking among college students in Botswana [22], a finding further supported by a study in a Hispanic community in New York City [23]. Social stigma fosters self-stigma through internalized negative stereotypes. Research from the United States and Canada indicates that higher self-stigma is associated with lower levels of help-seeking behavior [24]. Individuals experiencing self-stigma often feel embarrassed about seeking support. These findings suggest that the effects of self-stigma and social stigma on help-seeking may vary by context [24].

This current study addresses four questions: (a) What is the relationship between self-stigma and attitudes toward professional psychological help? (b) How does social stigma relate to attitudes toward seeking mental health services? (c) Do self-stigma and social stigma moderate the relationship between attitudes toward professional psychological help and intentions to pursue counseling? (d) Do experiences of anxiety and depression moderate the relationship between attitudes toward seeking professional psychological help and counseling-seeking intentions? This study was conducted in Ghana, a culture that values group cohesiveness and interdependence, where social dynamics may influence help-seeking behavior.

Although some studies in LMICs have examined the role of stigma and help-seeking behavior [25,26], there is still a need for more context-specific research, particularly in West African university settings. University students represent a unique group that faces emotional, academic, and social pressures, which may affect their willingness to seek psychological support [27]. In Ghana and other LMICs, mental health services are limited due to a shortage of trained professionals and restricted access to care [28,29]. To address these challenges, some Ghanaian universities have implemented student support services, such as counseling centers; however, utilization among students remains low [30]. While some studies have assessed the impact of stigma and locus of control on help-seeking attitudes in this context [31,32], few have explored the moderating effects of self-stigma and social stigma on help-seeking attitudes and intentions.

To address the gap in existing knowledge, this study examined the relationship between self-stigma and social stigma on help-seeking attitudes and counseling intentions, aiming to inform intervention strategies. We investigated the roles of self-stigma and social stigma as moderators of attitudes toward professional help and counseling intentions. We hypothesized that: (1) self-stigma will negatively correlate with attitudes toward professional help; (2) social stigma will negatively affect attitudes toward mental health services; (3) both self-stigma and social stigma moderate the link between attitudes and counseling intentions; and (4) anxiety and depression symptoms will moderate the relationship between attitudes and counseling intentions.

## Materials and methods

### Ethics statement

The Department of Psychology at the University of Ghana, through its Research and Ethics Committee, provided ethical approval for this study (Ethics Number: DREC/009/21–22). Respondents provided written informed consent before participating in the study.

### Participants

A total of 440 participants were recruited for this study through the distribution of questionnaires. Participation was voluntary, and questionnaires were distributed to readily available individuals in lecture halls and dormitories. Demographic information is reported for the full sample as shown in Table 1. The full sample consisted of 219 females (49.8%), 180 males (40.9%), and 41 individuals who did not disclose their gender (9.3%). The mean age was 22 years (*SD* = 3.57). There were some data entry errors that led to the accidental inclusion of 15-year-olds; these entries were removed prior to the analysis. Fourteen participants under the age of 18 were excluded from the main analysis to meet the age eligibility requirements, resulting in a final analytic sample of 426 participants. This final analytic sample included individuals aged 18–50 years, with a median age of 22 years; the majority were under 25 years old. Sixty participants were older than 25, demonstrating that the sample primarily consisted of young adults. All participants were enrolled in degree programs at two universities in Ghana.

**Table 1:** Sociodemographic characteristics of study participants (N = 440).

Variables	Frequency	Percentage (%)		
Gender				
Females	219	49.8		
Males	180	40.9		
Not reported	41	9.3		
**University level**				
100	94	21.6		
200	132	30.0		
300	116	26.4		
400	89	20.2		
Postgraduate	5	1.1		
Not reported	4	0.9		
Single	430	97.7		
Married/cohabit	6	1.4		
Not reported	4	0.9		
Age		Range	Mean	SD
	435	15 -50	22	3.57

### Procedure

We obtained ethical approval from the Department of Psychology at the University of Ghana through the Department’s Research and Ethics Committee (Ethics Number: DREC/009/21–22). Permission was also sought from the authorities at Koforidua Technical University. Data were collected from August 24, 2022, to November 11, 2022, following ethical approval. The questionnaires were given out by trained research assistants on the university campuses. It took about an hour to complete the pack of questionnaires. Participants were compensated with an amount of 10 cedis ($1.27). The exchange rate was based on rates during the data collection period.

### Measures

Our measures included measures of well-being, stigma, and attitudes and beliefs toward help-seeking. We considered it important to explore our attitude and intention variables separately. While attitudes reflect feelings and beliefs, intentions indicate the likelihood of engaging in a specific behavior. These often influence behavior differently.

The *Intentions to Seek Counseling Inventory *(ISCI)** [33] is a 17-item scale used to measure participants’ help-seeking intentions for various mental health problems. The scale consists of sample items such as “weight control” and “relational differences*.*” Participants indicate how often they will seek counseling for each item. Items were scored on a 4-point Likert scale (1 = very unlikely to 4 = very likely), with higher scores indicating greater willingness to seek counseling. The current sample reported an internal consistency of .85.

*The Attitude Toward Seeking Professional Psychological Help Scale (ASPH)* [6] is a 10-item instrument used to assess individuals’ attitudes toward seeking professional psychological help. It measures two dimensions: openness to seeking professional help for emotional problems and the perceived value of seeking professional help. Sample items in this scale include “I would want to get psychological help if I were worried or upset for a long period of time” and “a person should work out his or her own problems; getting psychological counseling would be a last resort.” The items are rated from 1 (disagree) to 4 (agree), with five items reverse-scored so that higher scores indicate more positive attitudes. The internal consistency of the measure for this sample was .60.

*Inventory of Attitudes Toward Seeking Mental Health Services (IASMHS)* [34] is designed to assess individuals’ attitudes toward seeking mental health services. It consists of 24 items rated on a 5-point Likert scale, with response options ranging from 0 (*disagree*) to 4 *(agree*). A higher score indicates a more positive attitude towards seeking help. The scale evaluates three factors: (a) psychological openness, (b) help-seeking propensity, and (c) indifference to stigma. Psychological openness reflects the extent to which individuals are open to acknowledging psychological problems and the possibility of seeking professional help. Help-seeking propensity reflects the extent to which individuals believe they are willing and able to seek professional psychological help. Indifference to stigma reflects the extent to which individuals are concerned about what various significant others might think if they find out that the individual is seeking professional help for psychological problems. Sample items in this scale include “psychological problems, like many things, tend to work out by themselves” and “having been mentally ill carries with it a burden of shame.” In this particular sample, the scale had an internal consistency coefficient (Cronbach’s alpha) of .67, following the removal of five items (items 1, 8, 13, 14, 23). Due to their low reliability, these items were excluded from the model computations, and only the summed scale (not subscale) was used for subsequent analyses.

*The Self-Stigma of Seeking Help Scale (SSOH)* [35] is a 10-item measure of self-stigma related to seeking psychological help. Self-stigma in this context refers to the fear that seeking help or attending therapy will reduce a person’s self-esteem, confidence in themselves and their abilities, and overall worth as a person. The self-scale consists of items such as “seeking psychological help will make me feel less intelligent” and “I would feel worse about myself if I could not solve my own problems.” Participants rated the items on a 5-point Likert scale from 1 (strongly disagree) to 5 (strongly agree). Higher scores indicate greater concerns about the impact of seeking help on self-esteem and self-worth. To ensure the reliability of the scale in this study, six items (1,2,4,5, 7, 10) were removed, resulting in an internal consistency coefficient (Cronbach’s alpha) of .67, as outlined in Table 2.

**Table 2:** Mean, standard deviation, skewness, and kurtosis for study variables.

Variable	N	Minimum	Maximum	Mean	SD	Skewness	Kurtosis	Cronbach alpha
ISC	396	15	60	40.02	8.80	-.32	.12	.85
ASPH	424	.00	30	15.80	4.72	-.48	1.03	.60
IASMHS	390	20	73	45.97	10.23	.31	-.44	.67
Self-stigma	430	3	20	8.85	3.55	.46	-.36	.67
Social stigma	434	.00	20	10.96	3.48	.16	-.09	.76

Note: ISC = Intentions to Seek Counseling; ASPH = Attitude Toward Seeking Professional Psychological Help; IASMHS = Inventory of Attitudes Toward Seeking Mental Health Services.

*The Stigma Scale for Receiving Psychological Help (SSRPH)* [36] is a 5-item social stigma scale designed to assess individuals’ perceptions of public stigma associated with seeking psychological treatment. The scale consists of items such as “seeing a psychologist for emotional or interpersonal problems carries social stigma” and “if people knew someone was getting help or advice, they would tend to like them less”. Participants rate each item on a 4-point Likert scale ranging from 0 (strongly disagree) to 3 (strongly agree), with higher scores indicating a greater perception of stigma linked to receiving psychological treatment. In our study, the SSRPH demonstrated good internal consistency, as indicated by a Cronbach’s alpha coefficient of.76, as detailed in Table 2. This scale measures public stigma — the negative attitudes, beliefs, and behaviors that motivate rejection and discrimination against individuals who seek psychological treatment.

*The State Trait Anxiety (STAI)* [37] is a 20-item self-report assessment employed in this study, serving as a reliable measure of anxiety and widely applied in both research and clinical contexts. Sample items in this scale include “I feel jittery” and “I feel worried.” Some of the items within this scale are reverse-coded. In our sample, the scale showed good internal consistency, with a Cronbach’s alpha coefficient of.76. Higher scores indicate a greater tendency to experience anxiety, whether in the moment or as a personality trait.

*The WHO-5 Well-Being Scale* [38] is a brief, self-administered measure of well-being over the past two weeks. It consists of five positively worded items, such as “I have felt cheerful and in good spirits” and “I have felt calm and relaxed”. These items are rated on a 6-point Likert scale, ranging from 0 (at no time) to 5 (all of the time). Higher scores indicate better well-being. The WHO-5 Well-Being Index (WHO-5) is used as a screening measure for depression [39]. In our sample, the scale showed good internal consistency, with a Cronbach’s alpha coefficient of.82.

### Data analysis

The data analysis was conducted using IBM SPSS (v.21). This analysis encompassed descriptive statistics and a thorough examination of normality assumptions, as indicated by measures of skewness and kurtosis. The results of the normality tests revealed that the skewness and kurtosis scores for each item fell within the acceptable range (-2 and 2), as defined by previous research [40]. The data for this sample were normally distributed and suitable for statistical analysis, as presented in Table 2. The moderating role of anxiety and depression on attitudes toward seeking psychological help and counseling intentions was not significant, and thus, those results will not be discussed in this manuscript. However, a moderation effect was observed for self-stigma and reported. To examine the effects of the moderation of stigma, we used PROCESS MACRO. The independent variable (attitudes towards seeking professional psychological help), moderators (self-stigma and social stigma), and the outcome (intentions to seek counseling) were included in the model. The interaction term was tested for significance, and simple slopes were plotted. A post hoc sensitivity analysis was performed using G*Power to assess the statistical power of the moderation analysis. In a sample of 400 participants, the analysis indicated that an effect size as small as f² = 0.025 could be detected. with adequate power (α = 0.05, power = 0.80). This indicates that the current sample size is sufficient for identifying moderate effects within the regression model.

## Results

### Relationship among variables

We conducted a correlational analysis to examine the interrelationships between all the study variables. The resulting correlation matrix, presented in Table 3 below, highlights the significant associations among the study variables, denoted by asterisks (*).

**Table 3:** Correlations among study variables.

Variable	1	2	3	4	5	6	7	8
1.Age	–							
2.Gender	-.15**	–						
3.Educ level	.23**	-.10*	–					
4.ISC	.01	.05	.01	–				
5.ASPH	-.01	.07	-.03	.16**	–			
6.IASMHS	-.06	.15**	.02	.15**	-.01	–		
7.Self-stigma	.14*	-.08	-.09	.09	.21**	.12*	–	
8.Social stigma	.08	-.09	-.03	-.08	.02	-.32**	.21**	–

Note: **p ≤ .001; *p ≤ .05; a measure of a continuous variable; Gender; 0 = males, 1 = females,2 = rather not say; Educ level = educational level, 1 = level 100, 2 = level 200, 3 = level 300, 4 = level 400, 5 = postgraduate; ISC = Intentions to Seek Counseling; ASPH = Attitude Toward Seeking Professional Psychological Help; IASMHS = Inventory of Attitudes Toward Seeking Mental Health Services.

### Association between stigma, attitudes toward seeking professional psychological help, and attitudes toward seeking mental health services

A simple regression analysis was conducted to find out whether self-stigma is significantly associated with attitudes toward seeking professional psychological help. The results showed that self-stigma (β = .21, p < .001) had a significant positive relationship with attitudes toward seeking professional psychological help, as shown in Table 4*.* We also examined whether social stigma was significantly associated with participants’ attitudes toward seeking mental health services. The results of the regression analysis showed that social stigma explained 10.1% of the variance (R² = .101, F (1, 370) = 41.71, p < .001). We found that social stigma (β = -.32, **p* *< .001) was negatively correlated with attitudes toward seeking mental health services, as shown in Table 4.

**Table 4:** Regression analyses of stigma variables on help-seeking attitudes.

*Variable*	*B*	*SE*	β	*t*	*95% CI*		*p*	*Outcome*
					LL	UL		
Constant	10.08	1.37		7.34	7.38	12.78	<.001	ASPH
Self-stigma	0.21	0.05	0.21	4.27	0.11	0.3	<.001	ASPH
Constant	64.68	1.70		37.98	61.34	68.04	<.001	IASMHS
Social stigma	-0.96	0.15	-0.32	-6.46	-1.26	-0.67	<.001	IASMHS

Note: dependent variables = Attitudes Toward Seeking Professional Psychological Help (ASPH); Inventory of Attitudes Toward Seeking Mental Health Services (IASMHS); CI = Confidence Interval; LL = lower limit; UL = upper limit.

### Moderation analysis

**Fig 1:**
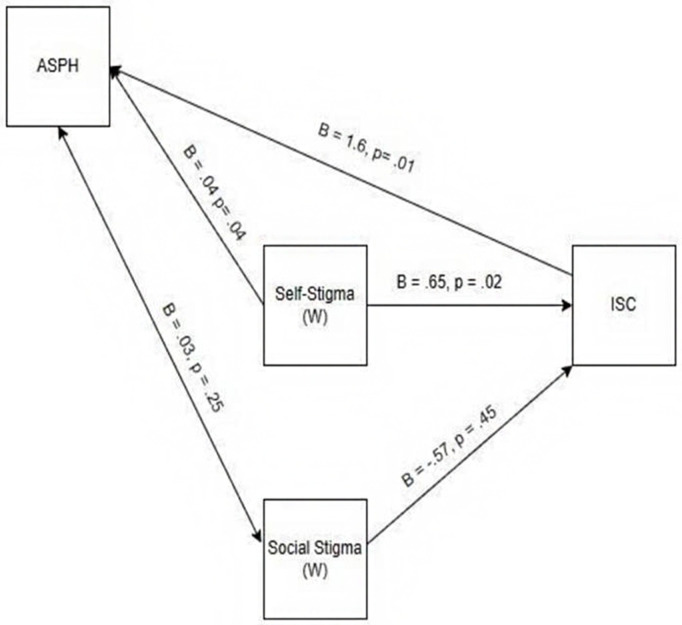
Observed model of the moderating roles of social stigma and self-stigma in the relationship between attitudes toward seeking professional psychological help and individuals’ intentions to seek counseling (ISC).

To address the question of the moderating effect of self-stigma and social stigma on attitudes towards seeking professional psychological help and intentions to seek counseling. We conducted a moderated regression analysis to test our hypothesis that the relationship between attitudes toward seeking professional psychological help (ASPH) and intentions to seek counseling (ISC) would be moderated by both social and self-stigmas. Our results are summarized in Table 5 and Fig 1.

**Table 5:** Moderated regression results of self-stigma on the relationship between attitude toward seeking professional psychological help and intentions to seek counseling.

Variables	B	SE *B*	*t*	*p-*value	*R*	*R* ^2^	95%	
							Lower	Upper
Constant	18.18	7.71	2.36	.02	.199	.039	3.01	33.36
ASPH	1.26	.47	2.66	.01			.33	2.20
Self-stigma	.65	.29	2.26	.02			.09	1.22
Self-stigma * ASPH	-.04	.02	-2.10	.04		.012	-.07	-.002

Note: ASPH = Attitudes Toward Seeking Professional Psychological Help; self-stigma was tested as moderator; constant = model intercept with Intentions to Seek Counseling as outcome.

Social stigma was not significantly associated with attitudes toward seeking professional psychological help and intentions to seek counseling services. Hence, there was no moderation effect of social stigma on attitudes toward seeking professional psychological help and intentions to seek counseling services.

We also tested self-stigma as a moderator between attitudes toward seeking professional psychological help and intentions to seek counseling. Self-stigma and attitude towards seeking professional psychological help collectively explained a significant portion of the variance in intentions to seek counseling (ISC) (R² = 0.039, F (3, 356) = 4.88, p = 0.002). The interaction term for self-stigma and attitude toward seeking professional psychological help (ASPH) was found to be significant (b = -0.04, p = 0.04). Hence, self-stigma moderated the relationship between attitudes toward seeking professional psychological help and intentions to seek counseling.

**Fig 2:**
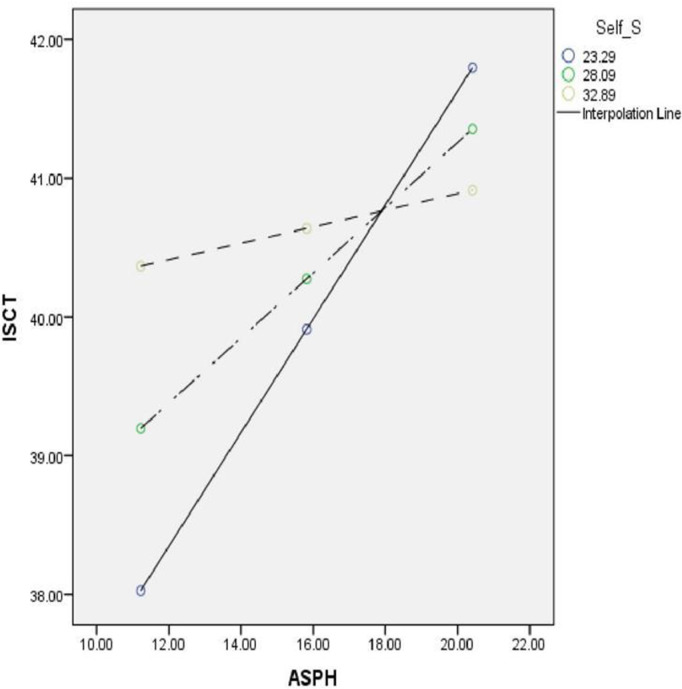
Simple slope analysis of self-stigma (self_s) moderating the relationship between attitude toward seeking professional psychological help (ASPH) and intentions to seek counseling (ISCT). Scores of 23.29 indicate low self-stigma, 28.09 indicate medium self-stigma, and 32.89 indicate high self-stigma.

A simple slope analysis, shown in Fig 2, was conducted to clarify the moderator effect (interaction). Our findings can be summarized as follows: At low levels of self-stigma and positive attitudes toward seeking professional psychological help, participants were more likely to express intentions to seek counseling. At moderate levels of self-stigma and positive attitudes toward seeking professional psychological help, participants still expressed their intentions, although at a reduced level compared to the low self-stigma group. Conversely, at high levels of self-stigma, participants scored much lower on intentions to seek counseling, even if they scored high on ASPH.

## Discussion

The main purpose of this study was to explore the factors associated with attitudes toward seeking mental health services, professional psychological help-seeking, and intentions to seek counseling among university students in Ghana. Here, we assessed factors influencing mental health-seeking behaviors, including self-stigma and social stigma.

Our findings revealed that self-stigma was significantly positively related to attitudes toward seeking professional psychological help, as measured by the Attitudes Toward Seeking Professional Psychological Help Scale. This finding is consistent with previous studies, including a qualitative study in Ghana [41] and a quantitative study in Lebanon [42]. This contradicts the common assumption that in collectivist cultures, where societal norms strongly impact social stigma, social stigma would be the main predictor of attitudes toward seeking professional psychological help.

Per our findings, social stigma had a significant inverse association with attitudes toward seeking mental health services as measured by the inventory of attitudes toward mental health services. This inventory measures an individual’s willingness to seek help not only from psychologists but also from other mental health professionals, such as psychiatrists, counselors, and social workers. We showed that high levels of social stigma are associated with poor attitudes toward mental health services, which is consistent with other previously published studies [43,44]. In the Ghanaian context, the lack of knowledge about mental health services, societal norms, and cultural beliefs perpetuates social stigma [45]. Additionally, the Ghanaian culture, known for its traditional beliefs in the supernatural causes of mental disorders, plays a significant role in shaping societal attitudes towards mental disorders [31]. These beliefs attribute mental disorders to curses and demonic possessions. Consequently, individuals tend to seek help from traditional healers rather than mental health professionals in the treatment process, further reinforcing the stigma associated with mental health services.

Our study showed that self-stigma, but not social stigma, moderated the relationship between attitudes toward seeking professional psychological help (ASPH) and intentions to seek counseling. Whereas social stigma may shape general attitudes, self-stigma may be a more psychologically potent factor affecting counseling-seeking intentions. Making sense of this finding involves exploring why an individual’s internalized negative beliefs and feelings about their mental health condition (self-stigma) would have a greater impact on the strength of the relationship between their attitudes and counseling intentions than the influence of broader societal perceptions and prejudices about mental health issues (social stigma). The difference must lie in the differential nature of social versus self-stigma. The previous finding of a positive relationship between self-stigma and ASPH, coupled with the moderating effect between ASPH and counseling-seeking intentions, suggests that the influence of self-stigma on help-seeking behavior, as far as mental health is concerned, is quite complex.

As discussed above, in our sample, individuals low on self-stigma with positive attitudes toward seeking professional psychological help were much more likely to intend to seek counseling than individuals high on self-stigma. A possible explanation for this finding could be an expectation that seeking counseling will inevitably require self-disclosure (of emotions, thoughts, and feelings) to a degree that people with high levels of self-stigma may be unwilling to engage in, even if they reported having positive attitudes towards psychological help-seeking in general. High levels of mental illness-related self-stigma imply that an individual ascribes to negative beliefs and stereotypes about themselves. These may fuel an unwillingness to open up about perceived signs of weakness in a counseling environment. Corrigan and Rao’s stage model of mental illness self-stigma suggests that once stigma is internalized, individuals experience diminished self-esteem and self-efficacy, exhibit hindered self-promotion behaviors, and may be socially avoidant [18]. All of these characteristics are consistent with low intentions to seek counseling.

## Limitations and conclusions

Although our study was conducted among a relatively large sample, it has a key limitation worth mentioning. Future research could explore the possibility of broadening the scope by including a wider range of universities in Ghana, expanding the study to universities in other countries, and exploring the relationship between the study’s variables in non-university samples. This would provide a deeper understanding of mental health-seeking behaviors. Additionally, some of our scales, including the Attitude Toward Seeking Professional Psychological Help Scale, the Inventory of Attitudes Toward Seeking Mental Health Services, and the Self-Stigma Scale of Seeking Help, had Cronbach’s Alpha values below .70. We acknowledge that these scales still provide valuable insights and have been utilized in similar studies [27,31]. Future studies should consider examining the reliability of these scales within a larger sample. Despite the limitations of this study, it adds to the limited literature on mental health help-seeking behaviors among university students in Ghana. In particular, discourse on mental health service provision in contexts such as those in which this study was conducted typically focuses on stigma in general and barriers to treatment, such as accessibility, resource availability, and religio-cultural barriers. The current study broadens the horizons of work in this area by identifying factors that predict students’ attitudes and intentions to seek professional psychological help. The identification of the differential roles of self-stigma and social stigma has some clinical implications.

Our findings suggest that optimizing mental health-seeking behavior in this and similar populations may warrant the development and testing of interventions that focus on addressing self-stigma directly rather than exclusively aiming to reduce mental health-related stigma at the societal level. Exploring culturally-tailored strategies to empower individuals to challenge and change their own negative beliefs and perceptions about themselves as far as their mental health is concerned may lead to improved self-esteem, agency, and willingness to seek help. However, achieving such a goal may be easier said than done. While self-stigma reduction interventions do exist (see, for example, a review by Yanos et al. [46], their designs have individual or group therapy/workshop formats, which could pose entry barriers since potential participants would have to be willing to attend such meetings to participate in the first place. Creating programming to address self-stigma that circumvents such entry barriers will thus require creativity, cultural sensitivity, and innovation. Adding such novel approaches to messaging that seek to counter existing social stigma and programming that aims to prevent social stigma will result in a multi-pronged approach to enhancing individual well-being, promoting supportive environments for those struggling with mental health difficulties, and possibly a reduction in premature, client-driven therapy terminations.
